# Veterinary Students’ Knowledge and Perceptions About Antimicrobial Stewardship and Biosecurity—A National Survey

**DOI:** 10.3390/antibiotics7020034

**Published:** 2018-04-18

**Authors:** Laura Hardefeldt, Torben Nielsen, Helen Crabb, James Gilkerson, Richard Squires, Jane Heller, Claire Sharp, Rowland Cobbold, Jacqueline Norris, Glenn Browning

**Affiliations:** 1 Asia-Pacific Centre for Animal Health, Department of Veterinary Biosciences, Faculty of Veterinary and Agricultural Sciences, Melbourne Veterinary School, University of Melbourne, Parkville, VIC 3050, Australia; helen.crabb@unimelb.edu.au (H.C.); jrgilk@unimelb.edu.au (J.G.); glenfb@unimelb.edu.au (G.B.); 2 National Centre for Antimicrobial Stewardship, Peter Doherty Institute, Grattan St, Carlton, VIC 3050, Australia; 3 School of Animal and Veterinary Sciences, University of Adelaide, Roseworthy, SA 5371, Australia; torben.nielsen@adelaide.edu.au; 4 College of Public Health, Medical and Veterinary Sciences, James Cook University, Townsville, QLD 4810, Australia; richard.squires@jcu.edu.au; 5 School of Animal and Veterinary Sciences, Charles Sturt University, Wagga Wagga, NSW 2650, Australia; jheller@csu.edu.au; 6 School of Veterinary and Life Sciences, Murdoch University, Perth, WA 6150, Australia; c.sharp@murdoch.edu.au; 7 School of Veterinary Science, University of Queensland, Gatton, QLD 4343, Australia; r.cobbold@uq.edu.au; 8 Sydney School of Veterinary Science, University of Sydney, Sydney, NSW 2006, Australia; jacqui.norris@sydney.edu.au

**Keywords:** education, antimicrobial resistance, personal protective equipment, antimicrobial stewardship, antibiotic

## Abstract

A better understanding of veterinary students’ perceptions, attitudes, and knowledge about antimicrobial stewardship and biosecurity could facilitate more effective education of future veterinarians about these important issues. A multicenter cross-sectional study was performed by administering a questionnaire to veterinary students expected to graduate in 2017 or 2018 in all Australian veterinary schools. Four hundred and seventy-six of 1246 students (38%) completed the survey. Many students were unaware of the high importance of some veterinary drugs to human medicine, specifically enrofloxacin and cefovecin (59% and 47% of responses, respectively). Fewer than 10% of students would use appropriate personal protective equipment in scenarios suggestive of Q fever or psittacosis. Students expected to graduate in 2018 were more likely to select culture and susceptibility testing in companion animal cases (OR 1.89, 95% CI 1.33–2.69, *p* < 0.001), and were more likely to appropriately avoid antimicrobials in large animal cases (OR 1.75, 95% CI 1.26–2.44, *p* = 0.001) than those expected to graduate in 2017. However, 2018 graduates were less likely to correctly identify the importance rating of veterinary antimicrobials for human health (OR 0.48, 95% CI 0.34–0.67, *p* < 0.001) than 2017 graduates. Students reported having a good knowledge of antimicrobial resistance, and combating resistance, but only 34% thought pharmacology teaching was adequate and only 20% said that teaching in lectures matched clinical teaching. Efforts need to be made to harmonize preclinical and clinical teaching, and greater emphasis is needed on appropriate biosecurity and antimicrobial stewardship.

## 1. Introduction

The association between antimicrobial use and increasing antimicrobial resistance (AMR) in animals has long been established [1,2,3,4,5,6,7,8,9,10]. Direct [11,12,13,14,15,16,17,18,19,20] or indirect [13,17,21,22] contact with animals can result in human–animal exchange of multidrug-resistant pathogens. Despite this established relationship, there is still widespread inappropriate prescribing of antimicrobials in all sectors of veterinary practice in Australia [23,24,25,26], indicating a need for antimicrobial stewardship (AMS) programs. Antimicrobial stewardship was conceptualized in the 1970s within the human health sector and has recently been defined as “a coherent set of actions which promote using antimicrobials responsibly” [27]. According to the World Health Organisation, “education of healthcare workers and medical students on rational antimicrobial prescribing or AMS is an integral part of all AMR containment activities” [28]. We would argue that education of veterinarians is equally important. 

Biosecurity also plays an important role in controlling antimicrobial resistance. In veterinary medicine, biosecurity is the set of preventative measures designed to reduce the risk of transmission of infectious diseases. The biosecurity habits of veterinarians have been investigated and 45% report contracting a zoonosis during their career and the reported use of personal protective equipment (PPE) was poor [29]. Equine veterinarians in Australia are reported as having up to 23-times-higher odds of carrying methicillin-resistant *Staphylococcus aureus* (MRSA) than controls [30], a trend that persists globally. Equine veterinarians who report use of PPE have 65% lower odds of carriage of MRSA compared with those not reporting PPE utilization [31]. There has been no investigation into veterinary students’ knowledge of and behavior regarding biosecurity or use of personal protective equipment.

Understanding the perceptions of veterinary students in different phases of their education is critical in guiding future education about AMS and resistance, in understanding the reasons for inappropriate antimicrobial use by recent graduates, and in guiding education about biosecurity measures. There is considerable literature on the knowledge and perceptions of medical students regarding AMS [32,33,34,35,36,37,38], but no studies to date on veterinary students. One United Kingdom study took a ‘One Health’ approach to this topic; however, the numbers of students from each sector responding to the survey were small and comparisons could not be made. Findings were largely consistent with previous research. Students recognized the global challenge of AMR but failed to recognize their personal prescribing practices as significantly contributing to the problem and mostly felt underprepared to prescribe antimicrobials appropriately [39]. To address the gap in understanding veterinary student knowledge and attitudes, we conducted a comprehensive survey of students in the last two years of their training across all Australian veterinary schools to assess the adequacy of current educational efforts and the factors influencing student attitudes and perceptions about AMS and biosecurity. 

## 2. Results

**Figure 1:**
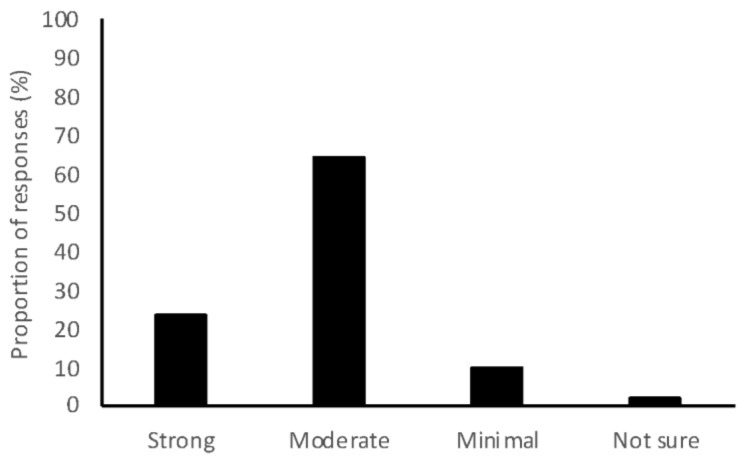
Proportion of 2017 and 2018 graduating Australian veterinary students (*n* = 476) responding to a survey indicating how much they think antimicrobial use by veterinarians contributes to the overall burden of antimicrobial resistance.

Of the 1246 Australian veterinary students graduating in 2017 or 2018, 476 (38%) completed the survey, with a further 30 responses incomplete and subsequently discarded. Students from all universities were represented and a significant sample was obtained from 6 of the 7 Australian veterinary schools (Table 1). Responses were obtained from 2017 graduates (227/476, 48%) and 2018 graduates (249/476, 52%). Students with varied interest areas completed the survey (Table 1). Overall, 88% of respondents thought veterinary use of antimicrobials had a moderate or strong contribution to overall AMR (Figure 1). Content analysis revealed the reasons for the contribution of veterinary antimicrobial use to overall AMR included overuse of antimicrobials (27% of respondents), overuse of antimicrobials in food animals (18% of respondents), and low use of culture and susceptibility (C & S) testing (7% of respondents). The residual variation due to university effects (variation partition coefficient) was 10–13% for outcomes of treatment and biosecurity, with most variance attributable to within-university and between-students. 

**Figure 2:**
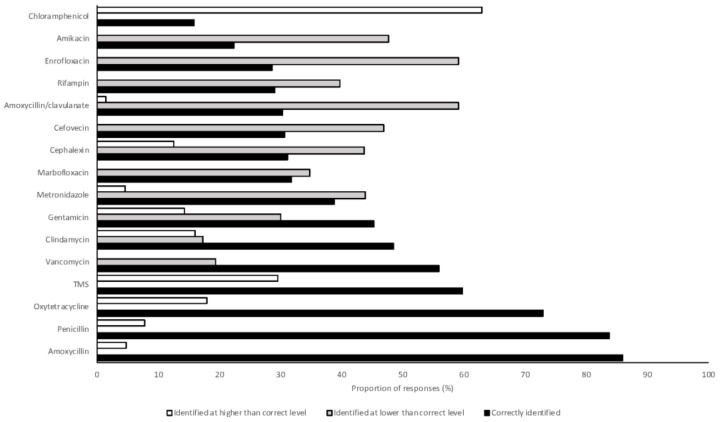
Proportions of 2017 and 2018 graduating Australian veterinary students (*n* = 476) responding to a survey correctly identifying the level of importance of antimicrobials in human medicine and identifying a level lower than or higher than that assigned by the Australian Strategic Technical Advisory Panel on Antimicrobial Resistance [40]. TMS, trimethoprim sulphonamide.

Over 80% of students correctly identified amoxycillin and penicillin as first-line therapies (86% and 84%, respectively) (Figure 2). All other antimicrobials were correctly categorized by fewer than 75% of respondents. Amoxycillin/clavulanate (second line) and enrofloxacin (third line) were incorrectly categorized into lower levels by 59% of respondents (for both drugs). Similarly, cefovecin (third line) was also commonly categorized into a lesser category (47% of respondents). Chloramphenicol (first line) was the only antimicrobial to be frequently categorized into a higher level (63% of respondents) (Figure 2). In the mixed effects model, 2018 graduates were significantly less likely to correctly identify the importance rating of antimicrobials than 2017 graduates, after adjusting for their area of interest and the random effect of place of study (OR 0.48, 95% CI 0.34–0.67, *p* < 0.001).

**Figure 3:**
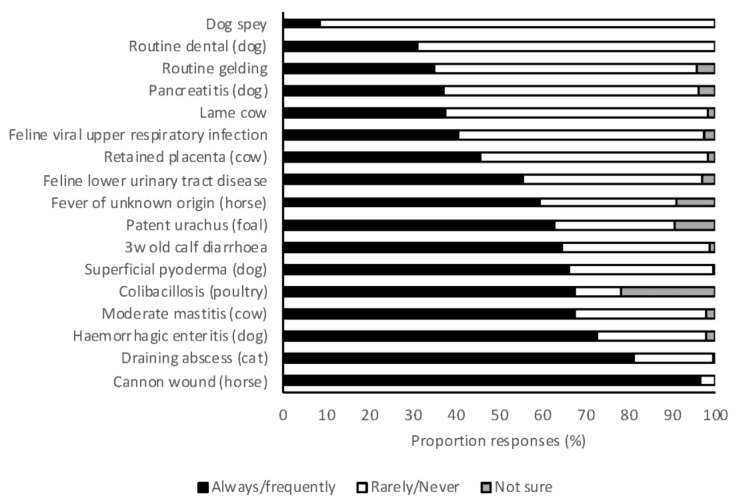
The frequency with which 2017 and 2018 graduating Australian veterinary students (*n* = 476) responding to a survey would treat a range of clinical scenarios with systemic antimicrobials.

Veterinary students were asked to indicate whether they would always, frequently, rarely, or never use systemic antimicrobials for a range of clinical scenarios. All scenarios were designed in such a way that systemic antimicrobials were rarely or never indicated. Always and frequently were combined, as were rarely or never, for ease of evaluation. Dog spey was the only scenario in which the vast majority of respondents indicated that antimicrobials were rarely or never indicated (91%) (Figure 3). After adjusting for their area of interest and the random effect of place of study, the 2018 graduates were significantly more likely to propose appropriate prescribing in large animal scenarios than 2017 graduates (OR 1.75, 95% CI 1.26–2.44, *p* = 0.001). In small animal scenarios, there was no difference in the appropriateness of prescribing by 2017 and 2018 graduates (OR 0.96, 95% CI 0.68–1.36, *p* = 0.83). There was no difference between students with small animal, mixed practice, or large animal practice interests in either small or large animal scenarios. 

**Figure 4:**
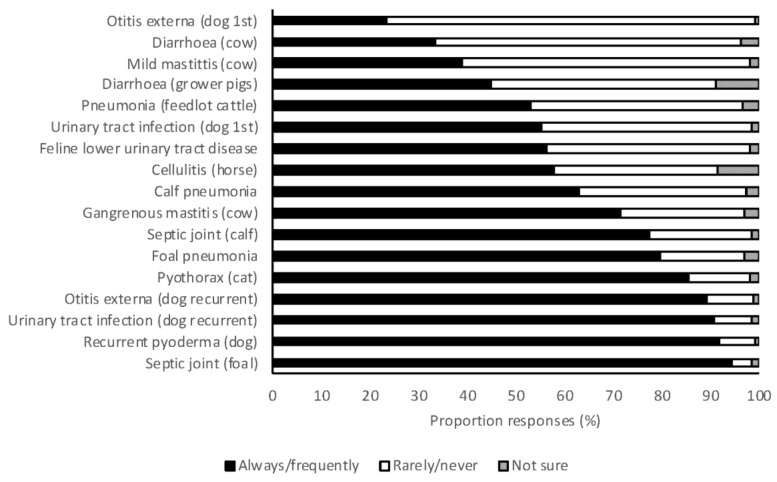
The frequency with which 2017 and 2018 graduating Australian veterinary students (*n* = 476) responding to a survey would perform culture and susceptibility for a range of clinical scenarios.

Veterinary students were asked to indicate whether they would always, frequently, rarely, or never utilize C & S testing for a range of clinical scenarios. At least 24% of students reported that they would perform C & S testing in each scenario, and at least 50% of students reported that they would always or frequently perform C & S in each of 13 of the 17 scenarios (Figure 4). Severe and recurrent infections were the scenarios most frequently associated with high rates of C & S testing. The most important factors that influenced students’ decisions to perform C & S testing were persistent infections (84%), recurring infections (71%), severe infections (38%), and client finances (32%). In small animal scenarios, 2018 graduates were significantly more likely to always or frequently perform C & S testing than 2017 graduates (OR 1.89, 95% CI 1.33–2.69, *p* < 0.001). There was no difference between year levels in large animal scenarios (OR 0.90, 95% CI 0.64–1.26, *p* = 0.537).

**Figure 5:**
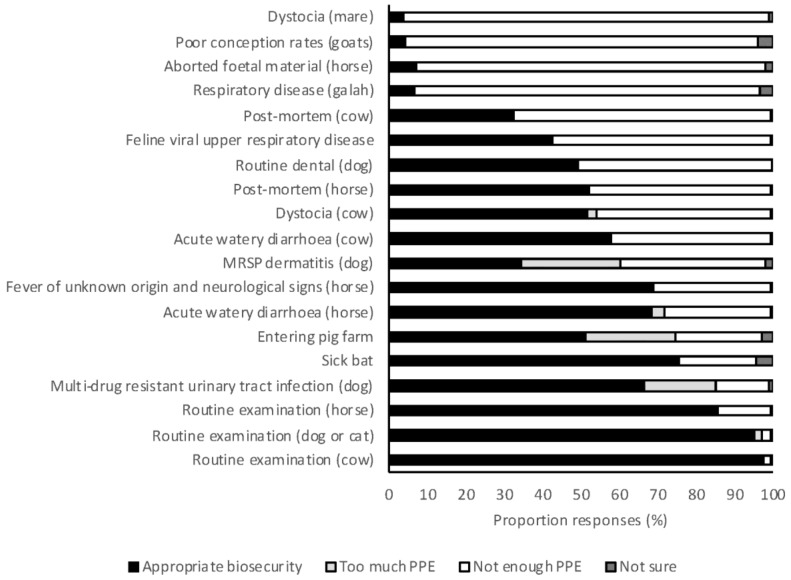
The frequency with which 2017 and 2018 graduating Australian veterinary students (*n* = 476) responding to survey applied appropriate biosecurity for a range of clinical scenarios. PPE, personal protective equipment; MRSP, multi-drug resistant *Staphylococcus pseudintermedius*

Appropriate use of PPE procedures and biosecurity were reported by the majority of students for routine examination of dogs and cats, cattle, and horses (97%, 98%, and 86%, respectively) (Figure 5). However, there were four scenarios in which the proposed use of PPE was insufficient for more than 90% of respondents: respiratory disease in a galah (*Elophus roseicapilla*), aborted fetal material from a horse, poor conception rates in goats, and dystocia in a mare (7%, 7%, 4%, and 4% of responses were appropriate, respectively). Students graduating in 2018 were significantly more likely to propose use of appropriate PPE in large animal scenarios than 2017 graduates (OR 1.15, 95% CI 1.02–1.29, *p* = 0.021). There was no difference in the appropriateness of use of PPE between year levels for small animal (OR 1.10, 95% CI 0.77–1.57, *p* = 0.606) or large animal scenarios (OR 1.39, 95% CI 0.97–1.99, *p* = 0.07). Students from the University of Sydney were significantly more likely to use appropriate PPE for large animal scenarios than students from all other universities (OR 1.23–1.55, *p* < 0.05 for all). This was largely due to an increased awareness of psittacosis, for which University of Sydney students were more likely to use appropriate PPE than students from other universities (OR 2.2–4.8, *p* < 0.05 for all). There were no differences between students based on area of interest.

**Figure 6:**
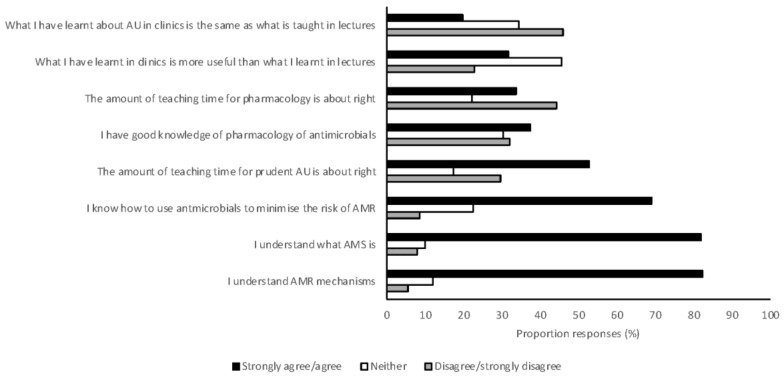
The opinion of Australian veterinary students, graduating in 2017 and 2018 and responding to a survey (*n* = 476), about the teaching of antimicrobial resistance and antimicrobial stewardship. AMR, antimicrobial resistance; AMS, antimicrobial stewardship; AU, antimicrobial use.

Students reported having a good understanding of the mechanisms of AMR and AMS (Figure 6). However, most respondents suggested that prudent antimicrobial use and pharmacology were taught less than was perceived necessary. Students also indicated that what they were taught in clinical practice about antimicrobial use was often different to what they were taught in preclinical learning activities (Figure 6). Students were largely aware of at least one of the antimicrobial prescribing guidelines currently available (83%), but rarely reported referring to these frequently (12%). Students had similar awareness of biosecurity guidelines, with 79% having at least heard of one of the biosecurity guidelines available for veterinarians in Australia. Biosecurity guidelines were also rarely referred to by students (3.5%). 

## 3. Discussion

To our knowledge, this is the first multicenter study investigating veterinary students’ knowledge and attitudes about AMS. This survey is also unique in its comparison of 2 years of veterinary students, gaining valuable insight into the progressive acquisition of AMS principles. Most students completing this survey thought that veterinary use of antimicrobials contributed moderately to the overall issue of AMR. This is consistent with a recent study of veterinarians in Australia [41]. There is little consensus between prescribing professions in Australia as to the proportional role of each in the evolution of AMR [42]. Students identified overuse of antimicrobials, especially in food animals, and low use of C & S, leading to inappropriate use of antimicrobials, as the main reasons underlying this contribution. In Australia, use of antimicrobials in food animals is modest in most industries, with most reports of multi-resistant organisms coming from companion animal species [20,43,44,45,46]. With evidence that both direct [11,12,13,14,15,16,17,18,19] and indirect [13,17,21,22] contact with animals can result in human acquisition of multidrug-resistant pathogens of animal origin, and high rates of pet ownership [47] in Australia, AMR in companion animal species is likely to pose the biggest risk to the community in Australia. Low use of C & S, due to the high cost of this testing, has been identified as a barrier to AMS in Australia [41]. As there is no obvious solution to this issue, rates of C & S testing are unlikely to increase. 

Ratings of the importance of antimicrobials in human health in Australia are assigned by the Australian Strategic Technical Advisory Group on AMR (ASTAG) [40]. Amoxycillin-clavulanate, enrofloxacin, and cefovecin were rated lower than by ASTAG by many students who responded to this survey (59%, 59%, and 47%, respectively). A recent survey of Australian veterinarians found that amoxycillin-clavulanate and cefovecin were frequently used in veterinary practice [48]. Antimicrobial use guidelines largely recommend amoxycillin alone as a first-line therapy and cefovecin is registered for use in Australia only after C & S testing [49]. Chloramphenicol was rated higher than by ASTAG by 63% of students in this survey. The adverse effects of chloramphenicol on people, and low use of this drug in clinical veterinary practice in Australia, may contribute to students erroneously believing that chloramphenicol has a higher importance rating. Students expected to graduate in 2018 were less likely to correctly identify the importance rating of antimicrobials than 2017 graduates (OR 0.48, 95% CI 0.34–0.67, *p* < 0.001), suggesting that greater exposure to use of antimicrobials during clinical teaching in the final year of veterinary school may lead to a greater awareness of antimicrobial importance ratings. 

In a recent survey, a high proportion of equine veterinarians [24] indicated that they would always or frequently use antimicrobials for an uncomplicated wound, and the survey described here also found that a very high proportion of students (97%) would always or frequently use antimicrobials for an uncomplicated wound over the cannon in a horse. Best-practice clinical guidelines do not recommend antimicrobials for uncomplicated wounds in horses [50,51]. Many students also indicated that they would always or frequently use antimicrobials for an uncomplicated draining abscess in a cat and hemorrhagic diarrhea in a dog (81% and 73%, respectively) even though guidelines recommend against such use [50,52]. However, some small animal textbooks [53,54] still recommend antimicrobials for hemorrhagic diarrhea, highlighting the need for harmonization of recommendations. Use of guidelines in preference to textbooks will also assist, as these should be more dynamic in their recommendations as an evolving evidence base arises. The rate of use of antimicrobials for abscesses in cats was assessed in a survey in 2011 and 52% (468/893) of respondents indicated that antimicrobials were not routinely prescribed (authors’ unpublished data). Students’ perceptions that use in these scenarios is warranted is concerning and efforts should be made to improve education in these areas. Students expected to graduate in 2018 were more likely to use antimicrobials appropriately in large animal scenarios than those expected to graduate in 2017 (OR 1.75, 95% CI 1.26–2.44, *p* = 0.001), suggesting that exposure to large animal clinical practice may be teaching students inappropriate antimicrobial use. In contrast, clinical experience did appear to improve student knowledge of the rating on antimicrobial importance, as described above. Contemporary research on the prescribing practices of Australian veterinarians showed that recent veterinary graduates were less likely to use antimicrobials appropriately than older graduates [23]. It was speculated that this may be due to inadequacies in preclinical and/or clinical university teaching, clinical teaching in the final year, or other challenges faced by recent graduates (greater fear of adverse events, workplace culture, or peer pressure). The hierarchical structure of veterinary practice has recently been identified as a barrier to effective AMS in veterinary practices [41]. This study suggests that universities should endeavor to promote AMS in both university teaching hospitals and associated extramural veterinary practices. 

Many students indicated they would always or frequently perform C & S testing in a range of scenarios in both large and small animal practice. Clearly there is a shift in behavior following the transition to clinical practice, as such high rates of C & S testing are not seen in clinical veterinary practice in Australia [26]. In small animal scenarios, 2018 graduates were more likely to perform C & S than 2017 graduates (OR 1.89, 95% CI 1.33–2.69, *p* < 0.001). Exposure to the low use of C & S in clinical practice may influence student decision-making in their final year of study. As seen in surveys of veterinarians in practice [41], severe and recurrent infections were most frequently recognized as always or frequently necessitating C & S testing. Restricted client finances were identified by 34% of practicing veterinarians as a barrier to C & S testing [41], and this was also recognized by students, with 32% indicating that this was an important factor in deciding whether or not to perform C & S.

There were four scenarios in which the vast majority of students indicated that they would use insufficient PPE. Two of these scenarios suggested commonly known zoonoses in Australian veterinarians. The first was avian psittacosis (respiratory disease in a galah), for which only 7% of respondents would use appropriate PPE, while the second was Q fever (poor conception rates in goats), for which only 4% of respondents would use appropriate PPE. The other two scenarios in which students indicated that they would use insufficient PPE described the recently identified risk of psittacosis after contact with fetal membranes in horses [55]. Personal protective equipment for Hendra virus infection in a horse was also concerningly inadequate given the wide publicity and educational effort given to this zoonosis in Australia (inadequate PPE selected by 31% of respondents). Students attending the University of Sydney were more likely to use appropriate PPE for the scenario suggestive of chlamydial abortion in horses. No differences were found between students attending different universities in their responses to the scenario suggestive of infection with Hendra virus. Hendra virus has caused fatal disease in veterinarians in Australia and this zoonosis has received much attention across the profession. Students should be aware of the measures needed to protect themselves, and others, from such zoonoses. Further studies are needed to clarify the methods used to convey this message to students and may be useful in providing effective means for conveying other key messages. 

Students in this survey frequently thought that more time should be spent on teaching pharmacology. Veterinary pharmacology is taught as a stand-alone subject in 5 of the 7 veterinary schools in Australia [56,57,58] and is integrated in the other courses. Similarly, surveys of medical students in the United Kingdom, France, and the United States of America have demonstrated that students also desire more education in this area [35,36,37,38]. Many students (45%) indicated that clinical teaching of antimicrobial use was not consistent with preclinical teaching, and many students (32%) felt that clinical teaching was more useful. However, students graduating in 2017, with higher levels of exposure to clinical teaching, had lower levels of compliance with guidelines compared with students graduating in 2018 before entering the clinical environment. This suggests that preclinical teaching is superior in the teaching of appropriate antimicrobial use. Efforts should be made to ensure consistency of teaching between preclinical and clinical teachers, and to ensure that appropriate AMS measures are in place in the clinical environment. Awareness of the existence of guidelines for antimicrobial use was high amongst this cohort of students, but utilization of these guidelines was low. Poor guideline utilization has been identified as an issue in medical hospitals [59], but the reasons are likely to be different for veterinary students. Understanding how guidelines are utilized in veterinary practice will allow for methods to be developed to optimize uptake. Malalignment of textbooks and current guidelines may contribute to differences in clinical behavior and further investigation is warranted.

Enrolment bias may occur with surveys such as this as respondents are self-selected. This factor may bias the results towards respondents that are more interested in AMS and therefore have more awareness of appropriate antimicrobial use. This survey had a good response rate, however, and high numbers of responses from 6 of the 7 universities. The results are unlikely affected by enrolment bias. Poor participation from one university was likely due to the differing graduation pattern at this school, with students graduating in August. For this reason, there were no 2017 graduate responses. While students in this survey reported having a good understanding of the mechanisms of AMR, the present study did not assess the understanding of AMR mechanisms. Further study is needed to confirm the perceptions of students.

## 4. Materials and Methods 

We conducted a cross-sectional multicenter study of the knowledge and attitudes of Australian veterinary students regarding AMS and biosecurity during October and November 2017. The source population comprised the national veterinary students expected to graduate in 2017 or 2018. All 2018 graduates had completed, or were near completion of, preclinical training but were yet to start the immersive clinical phase of training. All 2017 graduates had completed, or were near completion of, the clinical phase of training and were scheduled to graduate within 2 months of completion of the survey. Participation was voluntary and responses were anonymous. Students were recruited via email, social media, and by a researcher in class in some instances. Sample size calculations were performed to determine the number of respondents required to make appropriate inferences from the survey. To be 95% certain that our estimate of the population prevalence of veterinary students selecting a given treatment was within 5% of the true population prevalence, a total of 308 completed surveys were required. To detect a 15% difference between 2017 and 2018 graduates in the proportion answering correctly in any one part of the questionnaire, 388 completed surveys were required. To allow for comparisons between universities, 44–54 students from each university were required to complete the survey, which took into account the number of students in university year levels [60].

A questionnaire was developed using REDCap electronic data capture tools (Vanderbilt University, Nashville, TN, USA) [61] that consisted of 6 sections and 88 questions (available as Supplementary Materials). The initial section asked for demographic details about the respondents and their opinion of the degree to which veterinary antimicrobial use contributes to community AMR. Questionnaire Section 2 required respondents to indicate whether 16 named antimicrobials were first-, second-, or third-line therapies (as defined by the ASTAG) [40] with low importance rating agents classified first-line therapies, medium importance rating agents classified as second line, and high importance rating agents classified as third-line therapies). Section 3 required respondents to indicate the frequency (always, frequently, sometimes, rarely, never, or not sure) with which they would use antimicrobials for 17 specific scenarios (both medical and surgical). Section 4 required respondents to indicate the frequency (always, frequently, sometimes, rarely, never, or not sure) with which they would submit samples for culture and susceptibility (C & S) testing for 17 specific scenarios. Section 5 required respondents to indicate the level of biosecurity they would undertake for 19 specific scenarios. The final section required respondents to indicate their knowledge of common guidelines and their opinion about the quantity and quality of teaching on AMS within their program. All questions were closed except for one requesting the respondent’s opinion about the community impact of veterinary antimicrobial use. The survey was pretested with 2 recently graduated veterinarians and no changes were made.

The entire section of each part of the survey had to be completed by the respondent to be included in the analysis. Descriptive statistics were computed, with percentages reported for the proportion of the total number of respondents answering a particular question. Answers to questions that used a 5-point Likert scale were condensed into 3 categories (agree/strongly agree, neutral, disagree/strongly disagree). Differences in proportions were tested using χ^2^ test. An overall knowledge score was assessed for each section using antimicrobial use and biosecurity guidelines and by calculating the total proportion of correct answers.

A multilevel logistic regression model was used to identify individual student-level characteristics that were associated with correct identification of the importance rating of antimicrobials, appropriate antimicrobial usage, appropriate use of C & S testing, and correct use of PPE and biosecurity. Where students indicated they did not know the answer, these questions were discarded from the proportion of correct answers for this student. Results from the university with low numbers of responses were excluded from investigations of the effect of place of study. We started all analysis with a null model that included our binomial dependent variable and added the predictor variable of place of study to see whether the model was improved. Unconditional associations between each of the hypothesized explanatory variables (year level, veterinary school attended, species of interest) and the outcome of interest were examined using odds ratios. For the multivariable model, the outcome of interest was parameterized as a function of the explanatory variables. The random effect of university was tested in the model using a likelihood ratio test. Plausible two-way interactions were tested at an alpha level of 0.05. Where interactions were present, odds ratios are presented for each group. Analysis was performed using Stata, version 14.2 (StataCorp LLC, College Station, TX, USA).

Questions with open responses were openly coded and analyzed by one researcher (LYH) using content analysis and qualitative data analysis principles [62,63,64,65]. The code structure was developed using an inductive approach.

This research was approved by the University of Melbourne Faculty of Veterinary and Agricultural Sciences Human Ethics Advisory Group under Approval No. 1750016.1.

## 5. Conclusions 

In conclusion, this research has identified some gaps in the AMS education of veterinary students in Australia. Specifically, that the antimicrobials with a high importance rating that are in common use in veterinary practice should be identifiable by students. In addition, the lack of knowledge about appropriate use of PPE was concerning. Student perceptions and approaches indicate that preclinical AMS teaching is superior to clinical teaching and harmonization is recommended. Efforts are needed to improve guideline utilization by veterinarians, as this has been associated with more appropriate use of antimicrobials in human medicine [66,67] and in a veterinary teaching hospital [68]. Further research is needed into the barriers to usage and implementation of guidelines in the veterinary profession.

## Figures and Tables

**Table 1:** Demographics of 2017 and 2018 graduating Australian veterinary students (*n* = 476) responding to antimicrobial stewardship questionnaire.

Exposures	Number of responses (%)	University response rate, %
**University**		
Charles Sturt University (2018 graduates only)	7 (1.5)	11
James Cook University	55 (12)	37
Murdoch University	65 (14)	26
University of Adelaide	81 (17)	72
University of Melbourne	170 (36)	71
University of Queensland	53 (11)	22
University of Sydney	44 (9)	23
**Year of graduation**		
2017	227 (47)	
2018	249 (52)	
**Area of interest**		
Small animal	201 (42)	
Mixed practice	182 (38)	
Public health, government, industry, research	25 (5)	
Equine	23 (5)	
Bovine	18 (4)	
Undecided	27 (6)	

## Supplementary Materials

The following are available online at http://www.mdpi.com/2079-6382/7/2/34/s1. Questionnaire: Antimicrobial stewardship survey.
