# Supplementary material for: Veterinary Students’ Knowledge and Perceptions About Antimicrobial Stewardship and Biosecurity—A National Survey
Source: Antibiotics (Basel). 2018 Apr 18;7(2):34. doi: 10.3390/antibiotics7020034 (PMC6023091; doi:10.3390/antibiotics7020034)
Supplement: Supplementary file 1 [file antibiotics-07-00034-s001.pdf]

Antimicrobial stewardship survey

Please complete the survey below.
If you'd like more information about this survey click on the link to the participant information sheet.
[Attachment: "PLS_v1.docx"]

Which university do you attend?

- ◯ Charles Sturt University
- ◯ James Cook University
- ◯ Murdoch University
- ◯ University of Adelaide
- ◯ University of Melbourne
- ◯ University of Queensland
- ◯ University of Sydney

All going well, in which year will you graduate?

- ◯ 2017
- ◯ 2018
- ◯ 2019

What is your main area of interest?

- ◯ Companion animals
- ◯ Equine
- ◯ Bovine
- ◯ A mix of the above
- ◯ Public health, government, industry, research
- ◯ Haven't decided yet

Do you think that veterinary use of antimicrobials contributes to overall antimicrobial resistance?

- ◯ Strong contribution
- ◯ Moderate contribution
- ◯ Minimal contribution
- ◯ No contribution
- ◯ Not sure

For the question above, what are the major reasons you answered this way?

____

For each of the following antimicrobials, please indicate if they are for first, second or third line therapy:

	First line	Second line	Third line	Not sure
Amoxicillin	◯	◯	◯	◯
Amoxicillin clavulanate	◯	◯	◯	◯
Amikacin	◯	◯	◯	◯
Oxytetracycline	◯	◯	◯	◯
Metronidazole	◯	◯	◯	◯
Enrofloxacin	◯	◯	◯	◯
Procaine penicillin	◯	◯	◯	◯
Cefovecin	◯	◯	◯	◯
Trimethoprim sulphonamide	◯	◯	◯	◯
Gentamicin	◯	◯	◯	◯
Vancomycin	◯	◯	◯	◯
Clindamycin	◯	◯	◯	◯
Chloramphenicol	◯	◯	◯	◯
Cephalexin	◯	◯	◯	◯
Marbofloxacin	◯	◯	◯	◯
Rifampicin	◯	◯	◯	◯

For each of the following scenarios please indicate if you think systemic (injectable or oral) antimicrobials are indicated

	Always	Frequently	Rarely	Never	Not sure
Routine dog spey	○	○	○	○	○
Pancreatitis in a dog	○	○	○	○	○
2 year old cat with stranguria and haematuria	○	○	○	○	○
Lame cow	○	○	○	○	○
Routine gelding	○	○	○	○	○
2 day old wound over the canon bone of a horse with bone exposed	○	○	○	○	○
Cat with a draining abscess on its face	○	○	○	○	○
Routine dental prophylaxis in a dog	○	○	○	○	○
Upper respiratory tract disease of 4 days duration in an otherwise healthy cat	○	○	○	○	○
Dog with superficial bacterial dermatitis	○	○	○	○	○
3 week old calf with diarrhoea	○	○	○	○	○
Foal with patent urachus	○	○	○	○	○
Horse with fever of unknown origin	○	○	○	○	○
Dog with severe haemorrhagic gastroenteritis but not sepsis	○	○	○	○	○
Cow that calved 2 days ago that has retained foetal membranes	○	○	○	○	○
3 year old cow with moderate mastitis in 1 quarter	○	○	○	○	○
Colibacillosis in a poultry flock	○	○	○	○	○

For each of the following scenarios please indicate if you would submit samples for culture and susceptibility testing:

	Always	Frequently	Rarely	Never	Not sure
2 year old cat with stranguria and haematuria	○	○	○	○	○
5 year old female dog with stranguria and haematuria (first occurrence)	○	○	○	○	○
5 year old female dog with stranguria and haematuria that has previously been treated with amoxicillin clavulanate	○	○	○	○	○
4 week old foal with pneumonia	○	○	○	○	○
A group of 4 week old calves with pneumonia	○	○	○	○	○
4 year old cow with mild mastitis in 1 quarter	○	○	○	○	○
4 year old cow with gangrenous mastitis (black mastitis)	○	○	○	○	○
Otitis externa in a dog (1st occurrence)	○	○	○	○	○
Recurrent otitis externa in a dog	○	○	○	○	○
Adult cow with acute watery diarrhoea	○	○	○	○	○
Horse with distal limb cellulitis in a single leg	○	○	○	○	○
Calf with a septic hock joint	○	○	○	○	○
Foal with a septic hock joint	○	○	○	○	○
Pyothorax in a cat	○	○	○	○	○
Dog with recurrent pyoderma	○	○	○	○	○
Diarrhoea in grower pigs	○	○	○	○	○
Pneumonia in a group of feedlot cattle	○	○	○	○	○

What are the 3 most important factors that would influence your decision to submit samples for culture and sensitivity?

- ☐ Location of the infection
- ☐ Ease of obtaining a sample
- ☐ Persistent infection
- ☐ Severe infection
- ☐ Client finances
- ☐ Unusual infection
- ☐ Atypical cytology
- ☐ To confirm a diagnosis
- ☐ Notifiable diseases
- ☐ Herd problems
- ☐ Re-occurring infections
- ☐ Other

Please specify ______________________________

For each of the following scenarios please indicate the level of biosecurity you would take for examination and performing procedures

	None	Hand wash after contact	Gloves only	Gown/overalls only	Gloves & gown/overalls	Gloves, gown/overalls & face protection (respiratory mask and goggles)	Gloves, overalls with head protection, P2 respiratory mask and goggles	Not sure
Routine examination of a dog or cat	○	○	○	○	○	○	○	○
Routine examination of a horse	○	○	○	○	○	○	○	○
Routine examination of a cow	○	○	○	○	○	○	○	○
Horse with fever of unknown origin and neurological signs	○	○	○	○	○	○	○	○
Horse with acute watery diarrhoea	○	○	○	○	○	○	○	○
Cow with acute watery diarrhoea	○	○	○	○	○	○	○	○
Cat flu of 4 days duration in an otherwise healthy cat	○	○	○	○	○	○	○	○
Post mortem examination of a cow	○	○	○	○	○	○	○	○
Post mortem examination of a horse	○	○	○	○	○	○	○	○
Galah with respiratory disease	○	○	○	○	○	○	○	○
Aborted foetal material from a horse	○	○	○	○	○	○	○	○
Routine dental prophylaxis in a dog	○	○	○	○	○	○	○	○
Cow with dystocia (calving)	○	○	○	○	○	○	○	○
Sick bat	○	○	○	○	○	○	○	○
Goats with poor conception rates	○	○	○	○	○	○	○	○
Mare with dystocia (foaling)	○	○	○	○	○	○	○	○
Methicillin resistant Staphylococcus pseudintermedius dermatitis in a dog	○	○	○	○	○	○	○	○
An animal with a multi-drug resistant urinary tract infection	○	○	○	○	○	○	○	○
When entering a pig farm	○	○	○	○	○	○	○	○

Please indicate your level of knowledge about the following guidelines:

	Know of	Have read	Refer to often	Never heard of
AVA Guidelines for veterinary personal biosecurity	○	○	○	○
AIDAP Practical infection control guidelines	○	○	○	○
University of Melbourne Australian veterinary prescribing guidelines	○	○	○	○
AIDAP Antimicrobial prescribing guidelines for dogs and cats	○	○	○	○

Please indicate how strongly you agree to each of the following statements:

	Strongly agree	Agree	Neither agree nor disagree	Disagree	Strongly disagree
I understand what antimicrobial stewardship is.	○	○	○	○	○
The amount of teaching time for prudent antibiotic use is about right.	○	○	○	○	○
I understand antimicrobial resistance mechanisms.	○	○	○	○	○
I have a good knowledge of the pharmacology of antibiotics.	○	○	○	○	○
The amount of teaching time for pharmacology is about right.	○	○	○	○	○
I know how to use antibiotics to minimise the risk of antimicrobial resistance developing.	○	○	○	○	○
What I have learnt about antibiotic use in clinics is the same as what is taught to me in lectures.	○	○	○	○	○
What I have learnt in clinics is more useful than what I learnt in lectures.	○	○	○	○	○

Anything else you'd like to tell us?

____

Thank you for completing the survey! You're entitled to a copy of the guidelines as a thanks from us. If you didn't get your copy please email me at: laura.hardefeldt@unimelb.edu.au
